# Evaluation of heat stress and cumulative incidence of acute kidney injury in sugarcane workers in Guatemala

**DOI:** 10.1007/s00420-019-01426-3

**Published:** 2019-04-17

**Authors:** Jaime Butler-Dawson, Lyndsay Krisher, Hillary Yoder, Miranda Dally, Cecilia Sorensen, Richard J. Johnson, Claudia Asensio, Alex Cruz, Evan C. Johnson, Elizabeth J. Carlton, Liliana Tenney, Edwin J. Asturias, Lee S. Newman

**Affiliations:** 1 Department of Environmental and Occupational Health, Center for Health, Work, and Environment Colorado School of Public Health, University of Colorado, Anschutz Medical Campus 13001 E. 17th Pl., Ste. W3111, Aurora, CO 80045 USA; 2 Colorado Consortium on Climate Change and Human Health, University of Colorado, Anschutz Medical Campus, Aurora, CO USA; 3 Department of Environmental and Occupational Health, School of Public Health, University of Colorado, Anschutz Medical Campus, Aurora, CO USA; 4 Department Kinesiology and Health, University of Wyoming, Laramie, WY USA; 5 Department of Emergency Medicine, University of Colorado School of Medicine, Anschutz Medical Campus, Aurora, CO USA; 6 Division of Renal Diseases and Hypertension, School of Medicine, University of Colorado, Anschutz Medical Campus, Aurora, CO USA; 7 Pantaleon, Guatemala City, GT USA; 8 Division of Pediatric Infectious Diseases, School of Medicine, University of Colorado, Anschutz Medical Campus, Aurora, CO USA; 9 Center for Global Health, Colorado School of Public Health, Anschutz Medical Campus, Aurora, CO USA; 10 Department of Epidemiology, Colorado School of Public Health, University of Colorado, Anschutz Medical Campus, Aurora, CO USA; 11 Division of Pulmonary Sciences and Critical Care Medicine, Department of Medicine, University of Colorado School of Medicine, Anschutz Medical Campus, Aurora, CO USA

**Keywords:** Agricultural workers, Kidney injury, Hydration

## Abstract

**Objective:**

Agricultural workers worldwide exposed to heat stress could be at the risk of kidney injury, which could lead to chronic kidney disease of an unknown origin (CKDu). Hydration has been promoted as a key measure to reduce kidney injury. In the presence of a hydration intervention, the incidence of acute kidney injury (AKI) was calculated in a sugarcane worker population in Guatemala and several risk factors were evaluated.

**Methods:**

We measured kidney function at the beginning and end of the work shift at three time points in 517 sugarcane workers. We defined AKI as an increase in serum creatinine of 26.5 µmol/L or 50% or more from the pre-shift value. Associations between AKI and risk factors were examined, including interactions with hydration status.

**Results:**

The prevalence of dehydration post-shift (> 1.020 specific gravity) was 11% in February, 9% in March, and 6% in April. Cumulative incidence of AKI was 53% in February, 54% in March, and 51% in April. AKI was associated with increasing post-shift specific gravity, a dehydration marker, (OR 1.24, 95% CI 1.02–1.52) and with lower electrolyte solution intake (OR 0.94, 95% CI 0.89–0.99).

**Conclusions:**

Dehydration and insufficient electrolyte consumption are risk factors for AKI. However even well-hydrated sugarcane workers routinely experience AKI. While hydration is important and protective, there is a need to understand other contributors to risk of AKI and identify prevention strategies with these workers.

**Electronic supplementary material:**

The online version of this article (10.1007/s00420-019-01426-3) contains supplementary material, which is available to authorized users.

## Introduction

Daily heat stress and dehydration may be associated with recurrent acute kidney injury (AKI) among agricultural workers around the world (Roncal-Jimenez et al. 2015). Workers who experience recurrent episodes of AKI may be at substantial risk for developing chronic kidney disease of unknown origin (CKDu), a newly described global epidemic (Johnson et al. in press; Vos et al. 2017), specifically in agricultural communities in low altitudes along the pacific coast of Central America (Hahn et al. 2017; Roncal-Jimenez et al. 2015). Any workers who regularly labor in hot conditions could be at the risk of kidney injury and CKDu, with profound implications for worker health, safety, and sustainable food production as global temperatures continue to rise (Glaser et al. 2016; Watts et al. 2017).

There are a number of potential causes of AKI among agricultural workers that could operate independently or in combination. These include (1) volume depletion and dehydration due to heat stress and high physical demand, (2) sub-clinical or clinical rhabdomyolysis due to muscle damage from extreme labor, (3) tobacco use, and (4) non-steroidal anti-inflammatory drug (NSAID) use (Butler-Dawson et al. 2018; Correa-Rotter et al. 2014; Hodgson et al. 2017; Speeckaert et al. 2013). Agricultural work, specifically sugarcane cutting, involves intense labor in very hot temperatures (Butler-Dawson et al. 2018; Correa-Rotter et al. 2014; Crowe et al. 2013). Recent studies have shown heat stress to be a risk factor for cross-shift kidney damage in agricultural workers in Central America and the U.S. (Garcia-Trabanino et al. 2015; Mix et al. 2017; Moyce et al. 2017; Sorensen et al. 2019). Dehydration has also been identified as a risk factor for kidney damage among sugarcane workers laboring under hot conditions (Roncal-Jimenez et al. 2015). However, most studies have not found direct associations between dehydration and kidney injury (Butler-Dawson et al. 2018; Laws et al. 2016; Moyce et al. 2017).

There are few studies geared towards interventions to address the risk of repeat heat stress and dehydration in sugarcane worker populations. One study conducted in El Salvador implemented a water, rest, shade (WRS) intervention halfway through the 6-month sugarcane harvest (Bodin et al. 2016; Wegman et al. 2018). These authors reported that the WRS intervention resulted in a reduction in dehydration symptoms and a smaller decline in kidney function across the work shift compared to sugarcane workers not receiving the intervention. While the results suggest a protective effect of increased water consumption, direct associations between hydration and kidney function were not examined. Therefore, it remains unclear whether increasing water intake can reduce AKI events across the work shift among agricultural workers.

We implemented an enhanced hydration intervention among a large number of sugarcane workers in southwest Guatemala to evaluate the effect of improved hydration on cumulative incidence of AKI and to determine the role of other risk factors. We hypothesized that kidney injury, occurring at a sub-clinical level due to heat stress while performing intense labor in hot conditions, would be associated with risk factors like dehydration, physical workload, use of nephrotoxic medications such as NSAIDs, tobacco use, and sugary beverage intake (Correa-Rotter et al. 2014; Garcia-Arroyo et al. 2016; Speeckaert et al. 2013; Weiner et al. 2013).

## Methods

- Study population
  For this prospective longitudinal cohort study, we collected demographic, clinical, and laboratory data to evaluate kidney function and risk factors in 517 sugarcane workers from ten field work groups. Work groups consisted of sugarcane cutters and production workers employed for the 2016–2017 harvest season at a sugarcane mill in Guatemala owned by Pantaleon, an agribusiness and producer of sugar in Central America (Fig. 1). Cane cutters cut and pile sugarcane while production workers perform tasks such as cutting and planting cane seed. Work setting, worker population, and work practice details have been previously described (Butler-Dawson et al. 2018) and more detail is provided in the supplemental material.
  **Fig. 1:**
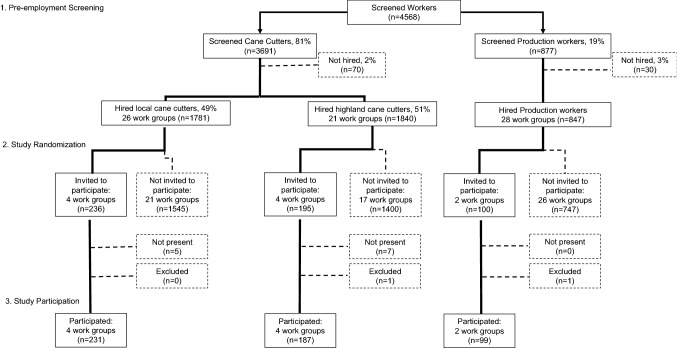
Flowchart of pre-employment screening and study recruitment
  All study participants (≥ 18 years) were male, screened for employment between August and November, and started the season with an estimated glomerular filtration rate (eGFR) ≥ 60 ml/min/1.73 m^2^ and no major illnesses that might affect their ability to work.
- Study design
  Data were collected before and after three work shifts for each work group during three consecutive months, February, March, and April 2017. In January 2017, prior to the start of data collection, workers from ten randomly selected work groups were recruited and consented for this study. We used stratified random sampling to construct the list of ten work groups stratified by job type, eight cane cutter work groups and two production work groups (Fig. 1). Among the cane cutter work groups, we also stratified by home residence. Four local resident work groups and four highland resident work groups were selected. Workers in the local resident work groups live in local communities in the coastal lowland area surrounding the sugarcane fields. Workers in the highland resident work groups are from the highland regions at higher altitudes, however, they live in dormitories at the mill for the duration of the harvest. Workers in the production work groups are only from the local communities.
  Data were available on baseline demographics for the worker population from which study participants were drawn. Pantaleon routinely collects pre-employment survey, clinical and laboratory data, including creatinine measures on the field workers, and we were able to leverage those data to assess differences between workers selected for the study and workers not selected. There were no significant differences in age (*p* = 0.49), home residence (*p* = 0.15), baseline BMI (*p* = 0.32), and baseline eGFR (*p* = 0.29) between selected workers and workers not selected.
- Study intervention
  One of the goals of our study was to facilitate actionable workplace policies and practices by determining how well we could prevent AKI by maximizing hydration. To accomplish this, we enhanced the current WRS program used by the company since 2009 (supplemental material). The enhanced WRS intervention was provided to all study participants and included two components: (1) amplifying the existing worker education program on the importance of WRS, and (2) providing a “wellness incentive” based on workers’ hydration status at the start and end of the work shift. At the time of consent and throughout the study, study personnel and trained Pantaleon nurse aides provided additional education through face-to-face communication, posters, and pocket urine color charts (Human Hydration, LLC, Hampton, VA) for self-evaluation of hydration status, translated to Spanish and adapted for low literacy (see supplemental material). For the wellness incentive, all study participants were offered incentives on their three study days. The worker received tokens if he started the study work shift hydrated (pre-shift urinary specific gravity was ≤ 1.020) (Perrier et al. 2017) or if he maintained or improved his hydration status across the work shift (< 1% body weight loss) (Webb et al. 2016). If a worker was not considered hydrated, he was encouraged to drink additional water and take more rest breaks. The workers entered tokens into a raffle for chances to win small non-monetary prizes (i.e., soap, towels, socks, soccer balls, plastic reusable food containers, and water bottles) at the end of each study day.
- Study ethics review
  Ethics review and approval for the study was done by the Colorado Multiple Institution Review Board (COMIRB) and in Guatemala by the Comite de Etica, Facultad de Medicina, Universidad Francisco Marroquin-Hospital Universitario Esperanza. All participants provided written informed consent at the time of enrollment.
- Data collection

### Pre-employment screening

To examine baseline health of the workers, we utilized pre-employment data, including creatinine measures that were collected by Pantaleon between August and November 2016. Serum creatinine was collected from a blood draw and sent to an independent, licensed clinical laboratory (Herrera Llerandi laboratory, Guatemala City, Guatemala). Creatinine values were performed in duplicate using the Creatinine Jaffe Generation 2 method. Survey data included number of previous harvests, self-reported diabetes, home residence, and job type. Clinical data included age, blood pressure (at least 3 min of seated rest before the measurement), weight, and height. Hypertension was defined as systolic blood pressure ≥ 140 mmHg and/or diastolic blood pressure ≥ 90 mmHg. We obtained written consent from study participants to link their pre-employment data to the study data.

### Pre- and post-shift sampling

Body weight, urinary specific gravity, and point-of-care (POC) creatinine were collected for each participant prior to the start of the work shift (5–9 am) at three time points. Body weight was measured using a scale (Microlife WS 100 digital scale, Clearwater, FL) that was placed on a stable platform and calibrated prior to each data collection session. Workers were weighed in work clothing with shin guards removed. To adjust for the additional weight of the clothes at the end of the day due to sweat and dirt, we calculated a correction factor (supplemental material) and subtracted the correction factor from the worker’s post-shift weight. Specific gravity was measured using a digital refractometer (ATAGO PAL-10S digital refractometer, Tokyo, Japan) within 10 min of workers providing a urine sample before and after the work shift, according to manufacturer’s instructions. To measure creatinine pre- and post-shift, blood was collected by finger prick and read instantly in the field using the Nova® Statscan (Stat Sensor Creatinine Meter, Nova Biomedical Corporation, Waltham, MA, USA).

The aforementioned pre-shift measures were repeated at the end of the work shift (1–4 pm for production workers and 4–7 pm for cane cutters). In addition, workers completed an interviewer-administered post-shift survey (supplemental material). The survey was piloted in a sub-sample of the target population and was delivered by Spanish-speaking interviewers. The survey included questions about workers’ past 24-h behaviors including heat- and pain-related symptoms during work shift (yes or no), number of cigarettes since they woke up, number of sugary beverages since they woke up, and number of alcoholic beverages consumed the previous night. The survey also included a detailed 24-h recall of oral and parenteral medications, vitamins, and supplements (yes or no and count for each type) and reasons why they took these items. We showed participants pictures of locally available pills and injectables as visual prompts. We created a variable, “NSAID use” (yes or no), based on the participant’s responses including use of ibuprofen, aspirin, diclofenac, naproxen, or local brands that were identified as an NSAID.

### Assessment of heat exposure

On each study day, we collected wet bulb globe temperature (WBGT) in the sugarcane field where the study group was working during work shift hours (3M QUESTemp 34, Thermal Environmental Monitor, St. Paul, MN, USA). WBGT is a measure of heat exposure and is used by the National Institute for Occupational Safety and Health (NIOSH) and the International Organization for Standardization (ISO) to establish guidelines for work/rest cycles in different environments (OSHA 2017). The WBGT meter provided output on the average and maximum WBGT for each study day. Of note, on the last study day in April, the WBGT meter only recorded for 40 min, from 2:00 to 2:40 pm. This 40-min WBGT measure could overestimate the average WBGT and potentially underestimate the maximum WBGT, so we excluded that day’s measurements.

### Assessment of acute kidney injury

The development of AKI was the primary health outcome variable. We identified workers with AKI based on their pre- and post-shift POC creatinine measures using the Kidney Disease: Improving Global Outcomes (KDIGO) criteria (KDIGO 2012). This criterion defines AKI as either an increase in the creatinine level by ≥ 26.5 µmol/L or an increase in the creatinine level to ≥ 1.5 times the pre-shift level. Based on previous comparisons between post-shift POC creatinine measures and venipuncture creatinine measures, we applied an adjustment factor of 0.7775 to all the POC creatinine values (Griffin et al. 2018). Adjusted POC creatinine was used to identify workers with AKI and calculate eGFR using the Chronic Kidney Disease Epidemiology Collaboration (CKD-EPI) equation for all participants (Levey and Stevens 2010).

### Assessment of hydration status and electrolyte solution intake

Hydration status was assessed in two ways: (1) percent change in body weight from pre- to post-shift, and (2) pre- and post-shift urine specific gravity. In addition, in the post-shift survey, we asked participants to quantify the number of liters of water and number of 500 ml electrolyte solution packets they had consumed since waking up that day.

### Assessment of physical work intensity

We measured workers’ levels of physical exertion using three variables: work shift hours, productivity, and productivity *z*-scores. The length of their work shift was based on the time the worker finished the pre-shift data collection and started the post-shift data collection. Productivity data were provided by Pantaleon on each cane cutter’s daily amount of cane they cut. These data included the number of tons of cane cut on each study day and the day prior to each study day for each cane cutter. Individual productivity *z*-scores were reported relative to an individual’s daily average tons cut during the entire season. The *z*-score was calculated using the following formula: (individual workers study day productivity – individual season average productivity)/individual standard deviation. Positive *z*-scores indicate above-average productivity, and negative scores indicate below-average productivity. In addition, participants reported the number of rest breaks (longer than 15 min), including lunch, that they took during the day’s shift.

f. Statistical analysis
  Participant characteristics (i.e., demographics, behavior, and clinical data) are described for both the cane cutters and production workers. The majority of these data were not normally distributed and are expressed as the median and interquartile range (IQR). Pre- and post-shift specific gravity were placed into three clinical categories (maximally dilute, < 1.005; hydrated, 1.005–1.020; and dehydrated, > 1.020) to present the hydration status across the 3 months.
  To address our hypothesis that AKI can be explained by certain risk factors, we estimated the associations between pre-specified risk factors and AKI using mixed-effects logistic regression analysis with all the workers combined. We began our statistical analyses by examining AKI in relation to each potential risk factor using linear mixed models with random intercept for each worker to account for repeated measures. We then examined correlations between WBGT and several variables that might be related to WBGT including work shift hours, post-shift specific gravity, electrolyte intake, and productivity using Spearman’s rank correlation coefficient (*r*). For the multivariable analysis, we included all variables that had *p* value < 0.10 in the univariate models and age, hypertension (yes or no), and baseline eGFR as covariates that were selected as a priori known risk factors for reduced kidney function. In the final multivariable model, both pre- and post-shift urinary specific gravity were included in the model, since they were only moderately correlated and not collinear (*r* = 0.42, *p* < 0.01). However, maximum WBGT and average WBGT were strongly correlated (*r* = 0.62, *p* < 0.001), so we ran the model with only average WBGT. Job type was significant in the univariate analysis; however, since job type was highly associated with specific gravity, one of our main exposures of interest, we did not include it in the final model.
  We were additionally interested in the extent to how dehydration modifies the relationship between certain behavioral risk factors and AKI. Based on previous literature, we examined the following risk factors: smoking (yes or no), sugary beverage intake (per 1 drink increase) and NSAID use (yes or no) (Butler-Dawson et al. 2018; Garcia-Arroyo et al. 2016; Lunyera et al. 2016). We fit a logistic regression model with an interaction term between each individual explanatory behavioral variable and post-shift urinary specific gravity on the odds of developing an AKI. We then included any significant interaction terms in the final multivariable model. A two-sided *p* value of < 0.05 was considered as the threshold for statistical significance. All the analyses were performed using SAS Release 9.4 (SAS Institute, Cary, NC).

## Results

### Study participation

Of 534 workers who were in the selected ten work groups, 522 were present the day of consent and enrolled in the study. Three workers who consented were not present on any of the study days and two workers were excluded from the analysis because they were supervisors and did not perform similar tasks. Therefore, we included 517 workers in this analysis. In total, 425 workers (82%) participated at all three time points, 66 workers (13%) participated at two time points, and 27 (5%) participated at one time point. In terms of study attrition, 32 workers (6%) dropped out of the workforce during the study, 54 (10%) were absent during their study day, and 10 workers (2%) refused to participate at one time point (8 in March and 2 in April). Participants who left the workforce early (28 cane cutters and 4 production workers) were significantly more likely to be younger (26 vs. 28 years, *p* = 0.04) and to be a highland worker compared to a local worker (10% and 4%, *p* < 0.01, respectively). Participants who were absent were more likely to be cane cutters than production workers (12% vs. 3%, *p* < 0.01). Participants who refused to participate were more likely to be highland workers than local workers (4% vs. 1%, *p* < 0.01). Study participants who left early, were absent, or refused were not significantly different in hypertension status or kidney function compared to those who remained in the study (supplemental material).

### Heat exposure

During the eight February study days, the average WBGT ranged from 29.5 to 32.9 °C and the maximum WBGT ranged from 31.7 to 36.4 °C. During eight March days, the average WBGT ranged from 28.2 to 32.2 °C and the maximum WBGT ranged from 31.2 to 35.5 °C. During the seven April days, the average WBGT ranged from 30.9 to 32.0 °C and maximum WBGT ranged from 33.5 to 35.5 °C. To put into context, the Occupational Safety and Health Administration (OSHA) heat exposure threshold for work/rest regimens for “very heavy” work at 30.0 °C is for workers to work 25% and rest 75% per hour (Crowe et al. 2013; OSHA 2017). Only 4 out of the 23 study days had an average WBGT below 30.0 °C.

- Cane cutter results.

### Baseline demographics and clinical data

In total, 418 cane cutters participated in the study. Cane cutters had a median age of 28 years (IQR 24–35) and a median BMI of 23 (IQR 21–25), Table 1. Fifty-five percent of the cutters were from the local area. Cane cutters reported having worked a median of seven harvest years (IQR 4–12). At baseline, 2% of the cutters had hypertension and none of them reported having diabetes.

**Table 1:** Cane cutter characteristics at three study time points in Guatemala, 2017

Pre-employment Screening	Characteristics, median (IQR), *n* (%)
Total number of workers	418
Baseline age, years	28 (24–35)
Baseline body mass index, kg/m^2^	22.6 (21.2–24.5)
Hypertension	8 (2%)
Home residence	
Local	231 (55%)
Highland	187 (45%)

Study time points	February	March	April
Participating workers (*N*)	411	355	373
**Hydration and electrolytes**			
Pre-shift body weight (kg)	59 (54, 64)	58 (54, 63)	58 (54, 63)
Body weight, percent change	0.3% (− 1.4, 1.8)	0.3% (− 1.6, 1.8)	1.1% (− 0.6, 2.8)
Pre-shift urinary specific gravity	1.003 (1.002, 1.006)	1.004 (1.002, 1.007)	1.004 (1.002, 1.008)
Maximally dilute (< 1.005)	279 (68%)	207 (58%)	188 (51%)
Normal (1.005–1.020)	116 (28%)	135 (38%)	177 (48%)
Dehydrated (> 1.020)	15 (4%)	13 (4%)	7 (2%)
Post-shift urinary specific gravity	1.002 (1.001, 1.004)	1.002 (1.001, 1.004)	1.002 (1.001, 1.004)
Maximally dilute (< 1.005)	335 (82%)	281 (79%)	290 (78%)
Normal (1.005–1.020)	68 (17%)	68 (19%)	74 (20%)
Dehydrated (> 1.020)	7 (2%)	7 (2%)	7 (2%)
Urinary-specific gravity, percent change^a^	− 0.07% (− 0.32, 0.07)	− 0.14% (− 0.43, 0.01)	− 0.10% (− 0.50, 0.00)
Electrolytes, number of 500 ml packets	6 (5, 8)	6 (5, 7)	6 (5, 8)
Water intake, liters	15 (15, 17)	15 (15, 17)	15 (15, 16)
**Physical work intensity**			
Rest breaks	4 (3, 4)	4 (3, 4)	4 (3, 4)
Work shift, hours	10.42 (9.90, 10.93)	10.90 (10.37,11.53)	10.45 (9.73, 11.10)
Productivity, prior day, tons/day	5.7 (4.6, 7.0)	5.3 (3.6, 7.4)	5.8 (4.8, 6.9)
Productivity, study day, tons/day	5.4 (4.3, 6.9)	6.0 (4.7, 7.9)	5.1 (4.1, 6.4)
Productivity *z*-score^b^, study day	− 0.20 (− 0.94, 0.44)	− 0.58 (− 1.12, − 0.05)	− 0.39 (− 0.81, 0.28)
**Heat exposure**			
Average WBGT, °C	32.5 (31.0, 32.5)	30.9 (30.7, 32.2)	31.3 (31.1, 31.4)
Maximum WBGT, °C	36.4 (33.8, 36.4)	35.1 (34.4, 35.1)	34.1 (33.7, 34.4)
**Study day behaviors**			
Smoked cigarette (count range)	12 (3%), (1–6)	13 (4%), (1–3)	19 (6%), (1–5)
NSAID use (count range)	32 (8%), (1–9)	9 (2%), (1–5)	15 (4%), (1–3)
Sugary drink consumption (count range)	99 (24%), (1–10)	89 (26%), (0.5–5)	138 (39%), (1–6)
Alcohol consumption (count range)	4 (1%), (2–5)	12 (3%), (1–4)	7 (2%), (1–7)
**Study day symptoms** ^c^			
Headache	13 (3%)	3 (1%)	11 (3%)
Fever	2 (< 1%)	0	2 (< 1%)
Urination pain	0	1 (< 1%)	1 (< 1%)
Vomit	0	1 (< 1%)	0
Weakness	0	2 (< 1%)	1 (< 1%)
Dry mouth	2 (< 1%)	2 (< 1%)	0
Swelling of hands and legs	1 (< 1%)	6 (2%)	0
Upper back pain	0	2 (< 1%)	2 (< 1%)
Lower back pain	5 (1%)	3 (1%)	3 (1%)
Breathing difficulty	4 (1%)	2 (< 1%)	2 (< 1%)

*NSAID* non-steroidal anti-inflammatory, *WBGT* wet bulb globe temperature

Hypertension, defined as systolic blood pressure ≥ 140 mmHg and/or diastolic blood pressure ≥ 90 mmHg

^a^Negative value shows a decrease in specific gravity across the work shift

^b^Z-score calculation: individual workers study day productivity—individual average productivity/individual standard deviation

^c^No participants reported the following symptoms: cramping in legs or arms, diarrhea, heart palpitations, dizzy, and ear pain

### Hydration status and electrolyte solution intake

As shown in Table 1, body weight was maintained across the work shift and percent change in body weight across the shift was slightly positive at all three points. Results from the pre-shift urinary specific gravity show that almost all of the workers started their work shift well hydrated (96% in February and March, and 98% in April). Notably, at the end of the shift, almost all of the workers had maintained hydration status. Workers reported drinking a median of three liters (six 500  ml packets) of electrolyte solution and drinking a median of 15 liters of water.

### Cumulative incidence of AKI and kidney function

Among the 418 cane cutters, 324 (78%) had at least one episode of AKI over the three work shifts. Thirty-eight percent of workers had one AKI, 28% of workers had two AKIs, and 11% had three AKIs. The cumulative incidence of AKI over a work shift was 47% in February, 51% in March and 45% in April (Table 2). The percent of workers with a stage 2 AKI doubled across the season, from 7 to 14%, defined as post-shift creatinine 2.0–2.9 times higher than pre-shift (KDIGO 2012).

The median eGFR at baseline was 116 ml/min/1.73 m^2^ and the median pre-shift eGFR was approximately 130 at all three time points. Almost all of the workers (92–94%) had a normal eGFR at the start of the work shift (≥ 90). One worker had an eGFR < 60 at all three time points and two workers had < 60 at two time points.

**Table 2:** Kidney function of the cane cutters at the three study time points in Guatemala, 2017

Kidney function	February	March	April
Pre-shift	*n* = 412	*n* = 355	*n* = 373
Creatinine, µmol/L, median (IQR)	59.23 (50.39, 71.60)	57.46 (47.74, 69.84)	57.46 (47.74, 68.95)
eGFR, ml/min/1.73 m^2^, median (IQR)	129.04 (118.20, 140.40)	130.87 (116.76, 142.42)	131.79 (118.99, 142.51)
Kidney disease stages, *n* (%)			
> 90 eGFR	380 (92%)	331 (93%)	348 (94%)
60–90 eGFR	25 (6%)	20 (6%)	19 (5%)
30–60 eGFR	5 (1%)	4 (1%)	5 (1%)
15–30 eGFR	1 (< 1%)	0	0

Cross-shift	*n* = 411	*n* = 348	*n* = 370
Change in creatinine, median (IQR)	0.27 (0.18, 0.38)	0.28 (0.18, 0.40)	0.27 (0.18, 0.38)
Acute kidney injury^a^, *n* (%)	191 (47%)	178 (51%)	166 (45%)
AKI categories^b^, *n* (%)			
Stage 1	176 (92%)	155 (87%)	140 (85%)
Stage 2	13 (7%)	20 (11%)	24 (14%)
Stage 3	2 (1%)	3 (2%)	2 (1%)

*eGFR* estimated glomerular filtration rate, *IQR* interquartile range

^a^Increase of post-shift creatinine by ≥ 26.5 µmol/L or 1.5 times pre-shift creatinine

^b^Stage 1: 1.5–1.9 times pre-shift or ≥ 26.5 µmol/L increase. Stage 2: 2–2.9 times pre-shift. Stage 3: 3 times pre-shift or 354 µmol/L increase

### Physical work intensity

Workers were in the field approximately 10 h at each time point, including rest periods. They reported taking a median of four rest breaks of at least 15 min duration. The median tons of cane cut by each worker on the study days were 5.7 tons in February, 5.3 tons in March, and 5.8 tons in April. The median tons cut on the day prior to the study day was 5.4 tons in February, 6.0 tons in March, and 5.1 tons in April.

### Behavioral risk factors and symptoms

Very few workers (< 9%) reported smoking any cigarettes the day of the study, taking a NSAID in the last 24 h, or consuming alcoholic beverages the night before. One-quarter of the workers reported drinking a sugary beverage the day of the study in February and March, with an increase to 40% in April. Very few of the workers reported symptoms on the study day, with headache being the most frequently reported symptom, at 3% in both February and April.

b. Production worker results

### Baseline demographics and clinical data

In total, 99 production workers participated. Production workers had a median age of 30 years (IQR: 24–38) and a median BMI of 24 (IQR: 21–26), Table 3. They reported having worked a median of five harvests (IQR: 2–9). At baseline, 9% had hypertension and no one reported having diabetes.

**Table 3:** Production worker characteristics at the three study time points in Guatemala, 2017

Pre-employment screening	Characteristics, median (IQR), *n* (%)		
Total number of workers	99		
Baseline age, years	30 (24–38)		
Baseline body mass index, kg/m^2^	24.0 (21.6–25.6)		
Hypertension	8 (9%)		
Study time points	February	March	April
Participating workers (*N*)	98	95	95
**Hydration and electrolytes**			
Pre-shift body weight (kg)	57 (54, 62)	59 (54, 64)	59 (54, 66)
Body weight, percent change	− 0.6% (− 1.6, 0.8)	− 0.6% (− 1.4, 0.3)	0.2% (− 0.9, 1.7)
Pre-shift urinary specific gravity	1.016 (1.01, 1.02)	1.014 (1.007, 1.019)	1.008 (1.004, 1.013)
Maximally dilute (< 1.005)	12 (13%)	13 (14%)	28 (29%)
Normal (1.005–1.020)	61 (64%)	59 (62%)	67 (71%)
Dehydrated (> 1.020)	23 (24%)	23 (24%)	0 (0%)
Post-shift urinary specific gravity	1.021 (1.014, 1.025)	1.014 (1.005, 1.024)	1.009 (1.002, 1.019)
Maximally dilute (< 1.005)	15 (15%)	21 (23%)	39 (41%)
Normal (1.005–1.020)	32 (33%)	38 (39%)	34 (36%)
Dehydrated (> 1.020)	51 (52%)	36 (38%)	22 (23%)
Urinary-specific gravity, percent change^a^	0.34% (− 0.14, 0.91)	0.16% (− 0.40, 0.69)	0.10% (− 0.40, 1.19)
Electrolytes, number of 500 ml packets	1 (0, 1)	1 (0, 1)	2 (2, 3)
Water intake, liters	5 (4, 6)	5 (4, 7)	5 (4, 6)
**Physical work intensity**			
Rest breaks	2 (1, 3)	2 (2, 3)	2 (2, 3)
Work shift, hours	8.00 (7.22, 8.65)	7.78 (7.38, 9.05)	7.92 (6.90, 8.18)
**Heat exposure**			
Average WBGT, °C	32.4 (29.5, 32.4)	29.9 (28.2, 29.9)	33.3 (32.0, 33.3)
Maximum WBGT, °C	34.7 (31.7, 34.7)	32.6 (31.2, 32.6)	33.8 (33.8, 35.2)
**Study day behaviors**			
Smoked cigarette (count range)	4 (4%) (1–10)	3 (4%) (1)	3 (4%) (1–3)
NSAID use (count range)	4 (4%) (1–3)	1 (1%) (1)	4 (4%) (1–2)
Sugary drink consumption (count range)	55 (56%) (1–3)	33 (38%) (1–3)	40 (48%) (1–3)
Alcohol consumption (count range)	3 (4%) (2–5)	3 (4%) (2–3)	1 (1%) (2)
**Study day symptoms** ^b^			
Headache	1 (1%)	1 (1%)	1 (1%)
Cramping in legs or arms	1 (1%)	0	0
Urination pain	1 (1%)	0	1 (1%)
Faint	1 (1%)	0	0
Dry mouth	9 (9%)	1 (1%)	1 (1%)
Upper back pain	2 (2%)	0	0
Lower back pain	3 (3%)	1 (1%)	1 (1%)

*NSAID* non-steroidal anti-inflammatory, *WBGT* wet bulb globe temperature. Hypertension, defined as systolic blood pressure ≥ 140 mmHg and/or diastolic blood pressure ≥ 90 mmHg

swelling of hands and legs

^a^Negative value shows a decrease in specific gravity across the work shift

^b^No participants reported the following symptoms: fever, diarrhea, heart palpitations, dizzy, vomit, ear pain, breathing difficulty, and

### Hydration status and electrolyte solution intake

As shown in Table 3, the percent change in body weight among the production workers was slightly negative in February and March and slightly positive in April. Results from the pre-shift urinary specific gravity show that 75% of these workers started their work shift hydrated in February and March. In April, all workers started the work shift hydrated. In notable contrast to the cane cutters, only half of production workers finished their work shift hydrated in February, 62% in March, and 77% in April. Workers reported drinking 500  ml to one liter of electrolyte solution and drinking a median of five liters of water.

### Cumulative incidence of AKI and kidney function

Among the 99 production workers, 97 workers (98%) had at least one episode of AKI. One-quarter of the workers (22%) had only one AKI, 35% had two AKIs, and 40% had three AKIs. AKI cumulative incidence across the work shift was 81% in February, 63% in March, and 77% in April (Table 4). The percentage of workers with a stage 2 AKI went from 30 to 16% across the study time points, coinciding with an improvement in availability of water at the later time point, as discussed below.

The median eGFR at baseline was 116 ml/min/1.73 m^2^ and the median pre-shift eGFR near 130 at all three time points. Over 90% of the workers had a normal eGFR at the start of the work shift (≥ 90). Four workers had a pre-shift eGFR < 60 and none of the workers had an eGFR < 60 at two or more time points.

**Table 4:** Kidney function of the production workers at the three study time points in Guatemala, 2017

Kidney function	February	March	April
Pre-shift	*n* = 98	*n* = 95	*n* = 95
Creatinine, µmol/L, median (IQR)	55.69 (42.43, 66.30)	56.58 (51.27, 68.95)	54.81 (47.74, 64.53)
eGFR, ml/min/1.73 m^2^, median (IQR)	133.14 (118.73, 147.35)	129.91 (113.18, 140.62)	130.60 (115.53, 143.27)
Kidney disease stages, *n* (%)			
> 90 eGFR	92 (94%)	86 (91%)	92 (97%)
60–90 eGFR	5 (5%)	6 (6%)	3 (3%)
30–60 eGFR	1 (1%)	3 (3%)	0
15–30 eGFR	0	0	0

Cross-shift			
Change in creatinine, median (IQR)	0.45 (0.33, 0.55)	0.34 (0.19, 0.44)	0.37 (0.29, 0.54)
Acute kidney injury^a^, *n* (%)	79 (81%)	60 (63%)	73 (77%)
AKI categories^b^, *n* (%)			
Stage 1	54 (68%)	56 (93%)	60 (82%)
Stage 2	24 (30%)	4 (7%)	12 (16%)
Stage 3	1 (1%)	0	1 (1%)

*eGFR* estimated glomerular filtration rate, *IQR* interquartile range

^a^Increase of post-shift creatinine by ≥ 26.5 µmol/L or 1.5 times pre-shift creatinine

^b^Stage 1: 1.5–1.9 times pre-shift or ≥ 26.5 µmol/L increase. Stage 2: 2–2.9 times pre-shift. Stage 3: 3 times pre-shift or 354 µmol/L increase

### Physical work intensity

Work shift hours were approximately eight hours at each time point, including rest breaks. Workers reported taking a median of two rest breaks that were more than 15 min.

### Behavioral risk factors and symptoms

Four percent or less of the workers reported smoking a cigarette the day of the study, taking a NSAID in the last 24 h, or consuming alcoholic beverages the night before. Over half of the workers reported drinking a sugary beverage on the day of the study in February, 38% in March, and 50% in April. Very few workers reported symptoms on the study days. In February, 9% of the workers reported dry mouth, 3% reported lower back pain, and 2% reported upper back pain. These symptoms decreased in March and April.

c. Analysis of risk factors

Table 5 summarizes the results of the univariate analysis. We found associations between AKI and several factors including age (odds ratio (OR) 1.01, 95% confidence interval (CI) 1.00–1.03), baseline eGFR (OR 0.98, 95% CI 0.98–0.99), pre-shift urinary-specific gravity (OR 1.41, 95% CI 1.19–1.67), post-shift specific gravity (OR 1.48; 95% CI 1.27–1.72), electrolyte solution intake (OR 0.89, 95% CI 0.85–0.93), rest breaks (OR 0.83, 95% CI 0.75–0.93), work shift hours (OR 0.89, 95% CI 0.83–0.95), job type (OR 3.10, 95% CI 2.29–4.19), and average and maximum WBGT (OR 0.90, 95% CI 0.82–0.98 and OR 0.89, 95% CI 0.82–0.96). Higher WBGT and more work shift hours were associated with a lower likelihood of developing an AKI. In a post hoc analysis, we examined this relationship and other possible factors that may be correlated with WBGT, including work shift hours, post-shift specific gravity, electrolyte intake, and productivity. Work shift hours were inversely associated with WBGT for cane cutters (as WBGT increased, work shift hours decreased: *r* = − 0.31, *p* < 0.01). Electrolyte solution intake was positively correlated with WBGT for both groups (as WBGT increased, electrolyte intake increased: cane cutters *r* = 0.28, *p* < 0.01 and production workers, *r* = 0.73, *p* < 0.01). Post-shift specific gravity and productivity were not correlated with WBGT.

**Table 5:** Mixed-effects logistic regression univariate analyses assessing the odds of acute kidney injury in sugarcane workers in Guatemala, 2017 (*N* = 517)

Characteristics	OR (95% CI)	*p* value
**Baseline**		
Age	1.01 (1, 1.03)	**0.04**
Local home residence (ref: Highland)	1.13 (0.89, 1.44)	0.32
Body mass index	0.99 (0.95, 1.04)	0.75
Hypertension (ref: no)	1.37 (0.68, 2.77)	0.37
Previous harvests	1.01 (0.99, 1.03)	0.45
Baseline eGFR	0.98 (0.98–0.99)	< **0.01**
**Hydration and electrolytes**		
Body weight, percent change	1.01 (0.98, 1.04)	0.55
Pre-shift specific gravity (per 0.01)	1.41 (1.19, 1.67)	< **0.01**
Post-shift specific gravity (per 0.01)	1.48 (1.27, 1.72)	< **0.01**
Specific gravity, percent change	1.07 (0.93, 1.22)	0.34
Electrolyte solution intake (per 1 packet)	0.89 (0.85, 0.93)	< **0.01**
**Physical work intensity**		
Rest breaks (per 1 break)	0.83 (0.75, 0.93)	< **0.01**
Work shift hours (per 1 h)	0.89 (0.83, 0.95)	< **0.01**
Productivity, prior day (per 1 ton) ^a^	1.00 (0.94, 1.06)	0.90
Productivity, study day (per 1 ton) ^a^	1.00 (0.95, 1.06)	0.93
Productivity *z*-scores (per 1 std. deviation)	0.93 (0.82, 1.05)	**0.25**
Production job type (ref: cane cutter)	3.10 (2.29, 4.19)	< **0.01**
**Heat exposure**		
Average WBGT (per 1 °C)	0.90 (0.82, 0.98)	**0.02**
Maximum WBGT (per 1 °C)	0.89 (0.82, 0.96)	< **0.01**
**Study day behaviors**		
Sugary beverage intake (per 1 drink)	1.06 (0.9, 1.24)	0.48
Smoked cigarette (ref: no)	1.36 (0.79, 2.35)	0.27
NSAID use (ref: no)	1.44 (0.84, 2.47)	0.18
Alcohol intake (per 1 drink)	0.95 (0.76, 1.19)	0.66

Bold values are significant at *p* < 0.05

*OR* odds ratio, *CI* confidence interval, *eGFR* estimated glomerular filtration rate, *WBGT* wet bulb globe temperature, *NSAID* non-steroidal anti-inflammatory drug

^a^Cane cutters only, *n* = 418

Table 6 shows the overall associations between examined risk factors and AKI in the multivariable regression model. We observed that higher post-shift specific gravity was associated with greater odds of developing an AKI (OR 1.24, 95% CI 1.02–1.52). Higher baseline eGFR and higher electrolyte solution intake were associated with lower odds of developing an AKI (OR 0.98, 95% CI 0.97–0.99 and OR 0.94, 95% CI 0.89–0.99, respectively). Higher pre-shift specific gravity approached statistical significance (OR 1.21, 95% CI 0.99–1.46). Next, we explored interaction terms independently to assess the compounding effect of dehydration and certain behaviors that have been hypothesized as contributors to kidney function decline; these included sugary beverage intake, smoking, and NSAID use. Only the interaction term between post-shift specific gravity and NSAID use was significant. Workers who used NSAIDs and had increasing specific gravity were 8.38 times more likely to experience an AKI (OR 8.38, 95% CI 1.67–42.16). The confidence interval is wide for this interaction term due to the low number of workers that reported using NSAID and the low number of workers with elevated specific gravity. The separate interaction terms with post-shift specific gravity and smoking and sugary beverage intake were not significant (OR 1.26, 95% CI 0.48–3.29 and OR 0.92, 95% CI 0.75–1.14, respectively). When we included the NSAID x post-shift specific gravity in the multivariable model, this interaction term remained significant, as did baseline eGFR and electrolyte solution intake (OR 6.05, 95% CI 1.49–24.62, OR 0.98, 95% CI 0.97–0.99, and OR 0.94, 95% CI 0.88–0.99, respectively). Pre-shift specific gravity became significant in this model and post-shift specific gravity approached significance (OR 1.21, 95% CI 1.00–1.46 and OR 1.20, 95% CI 0.98–1.47). While the interaction term is significant, there is high uncertainty in the estimate due to the wide confidence interval.

**Table 6:** Mixed-effect multivariable regression analyses showing odds ratios (95% CI) for risk factors for acute kidney injury in sugarcane workers in Guatemala, 2017 (*N* = 517)

Covariates	OR (95% CI)	*p* value
Age	0.99 (0.97–1.01)	0.22
Hypertension (ref: no)	1.10 (0.56–2.16)	0.79
Baseline eGFR	0.98 (0.97–0.99)	< **0.01**
Work shift hours	0.98 (0.90–1.06)	0.60
Rest breaks	0.99 (0.86–1.14)	0.94
Electrolyte solution (per packet)	0.94 (0.89–0.99)	**0.04**
Pre-shift specific gravity (per 0.01)	1.21 (0.99–1.45)	0.05
Post-shift specific gravity (per 0.01)	1.24 (1.02–1.52)	**0.03**
Average WBGT (per 1 °C)	0.93 (0.81–1.06)	0.25

Bold values are significant at *p* < 0.05

*CI* confidence interval, *eGFR* estimated glomerular filtration rate, *WBGT* wet bulb globe temperature

## Discussion

In this study, we evaluated 517 Guatemalan sugarcane workers to understand the effects of hydration, high physical workload, heat exposure and behavioral risk factors on AKI. A key discovery is that we observed a surprisingly high incidence of cross-shift AKI even when workers were well hydrated. Despite appropriate hydration in the field, increasing post-shift specific gravity was observed to be a risk factor for AKI and higher electrolyte solution intake was found to be protective. The fact that 81% of these sugarcane workers demonstrated evidence of at least one AKI during the study period demonstrates a higher vulnerability than has previously been reported in agricultural workers from the U.S. (Mix et al. 2017; Moyce et al. 2017).

These findings have practical public health implications. First, enhanced hydration, including use of electrolyte solution, is important to mitigate the risk of AKI in agricultural workers. Additionally, other factors appear to contribute to AKI risk in those who are dehydrated. Increasing post-shift specific gravity in combination with NSAID use was found to be a significant risk factor for AKI across the work shift. Therefore, the effect of NSAID use on AKI appears to be dependent on hydration status; however, these findings should be interpreted with caution due to the small overall presence of these events. These data support, but do not prove, the hypothesis that dehydrated individuals are at greater risk of harm from nephrotoxins (Correa-Rotter et al. 2014; Weiner et al. 2013).

While the present study did not find a direct association between increasing WBGT and AKI, heat stress cannot be ignored, since WBGT was over 30 °C on almost all study days with little variation. As expected, we observed that as WBGT increased, cane cutters shift hours decreased. This may explain why increasing WBGT was not associated with AKI. We used a single point measure, field-based WBGT, for the all the workers on each study day. Future studies should measure individual-level heat exposure. Several other studies have also observed cross-shift increase in serum creatinine under conditions of high heat stress and dehydration (Garcia-Trabanino et al. 2015; Mix et al. 2017; Moyce et al. 2017). A study in El Salvador, among 189 sugarcane cutters, found a significant increase in serum creatinine across the work shift which was positively associated with temperature and heat strain (Garcia-Trabanino et al. 2015). In the U.S., the relationship between AKI and heat stress has also been investigated. One study conducted in California with 283 agricultural workers observed a 12% incidence of AKI over a work shift and found a link between kidney injury and temperature (Moyce et al. 2017). Workers who experienced heat strain, evaluated on core body temperature and heart rate, had 34% increased risk of AKI. In a study conducted in Florida, 33% of the 192 agricultural workers had at least one AKI. For each 5 degree °F increase in heat index, odds of an AKI increased 37% (Mix et al. 2017). These workers had much higher pre- and post-shift urinary specific gravity than the workers in our study, with 53% of their participants demonstrating a pre-shift specific gravity ≥ 1.020 and 81% demonstrating a post-shift specific gravity ≥ 1.020.

In addition, we found that workers who reported higher electrolyte solution intake were less likely to develop an AKI. A study conducted in Nicaragua found that for every additional electrolyte solution packet consumed by cane cutters, mean late-harvest eGFR was 6.1 ml/min/1.73 m^2^ higher (Laws et al. 2015). One electrolyte of particular interest is potassium. For example, decrease in serum potassium levels across the work shift have been observed in agricultural workers with heat stress and dehydration (Garcia-Trabanino et al. 2015). In addition to potassium, changes in serum sodium may play a role in the development of AKI. A study conducted in Brazil among sugarcane workers found a negative correlation between the changes in serum creatinine and the changes in serum sodium across the work shift (Santos et al. 2015). Authors suggest that decrease in sodium could worsen volume depletion due to a decrease in plasma osmolality, which may explain why electrolyte solution appears to confer a protective effect against kidney injury.

Our study has several strengths. It is the first study that examines cross-shift creatinine on three workdays distributed throughout the harvest season in such a large cohort of workers. Second, we measured clinically important biomarkers for hydration status and kidney function and collected detailed information on risk factors at three separate time points. In addition, we were able to optimize hydration status among workers through an enhanced WRS intervention. Nevertheless, several limitations need to be acknowledged. Based on our previous study comparing post-shift POC creatinine to venipuncture measures, we applied a correction factor to pre- and post-shift POC creatinine measures (Griffin et al. 2018). Further work is required to determine if the correction factor is accurate for pre-shift measures. There are several other potential mechanisms for the development of AKI that were not studied, such as rhabdomyolysis and inflammation. In addition, there are other potentially nephrotoxic substances that we did not study for their contributions to AKI risk (Johnson et al. in press). Although it is theoretically possible that exertional rhabdomyolysis may have contributed to an increase in post-shift serum creatinine and affected our estimate of AKI, we think this is unlikely for three reasons. First, if rhabdomyolysis was a main contributor to AKI, it would have been expected that individuals that were more productive (i.e., cut more cane) would have incurred higher levels of muscle damage and therefore would demonstrate higher post-shift serum creatinine. This was not the case. Second, in a separate study, we measured post-shift serum creatine kinase (CK) in a subset of this current study population, 105 sugarcane cutters, during the same three time points, and observed only 2.5- to threefold increase of CK above baseline (Sorensen et al. 2019). In that group, there was no statistically significant association between CK level and post-shift serum creatinine. Third, in a study of 203 healthy individuals with exercise-induced elevation of CK, Clarkson et al. observed no rise in serum creatinine, despite CK levels 3.1- to 6.2-fold above baseline, at 4, 7, and 10 days post-exercise (Clarkson et al. 2006).

We acknowledge that there are more sensitive and precise indicators of physical exertion, such as heart rate, that can be used instead of tons cut and work shift hours. In an effort to improve precision, we calculated each worker’s productivity *z*-score to compare their study physical exertion to their average level of exertion.

An additional limitation is that it can be difficult to determine if workers are truly hydrated using specific gravity, since as kidney function declines, urine concentrating capacity is reduced (Zittema et al. 2017). We were unable to measure exactly when the fluid was consumed. While the specific gravity is surprisingly low in this group of workers, the low urine-specific gravity reflects the high water intake and is supported by the presence of other measures of hydration that were collected, and by absence of weight loss. Cane cutters reported drinking a median of 15 liters of water per day; these workers had very low specific gravity. Production workers reported drinking a median of 5 L of water per day and appropriately had higher specific gravity. Specific gravity was also measured using urine dipsticks for all participants and yielded very similar results compared to refractometer measures. Among the workers with a refractometer specific gravity of < 1.005, 98% had a dipstick specific gravity of less than or equal to 1.010. In our separate study of 105 sugarcane cutters (Sorensen et al. 2019), by calculating serum osmolality, we found that hypo-osmolality was common, with more than 30% of workers demonstrating a serum osmolality < 280 mmol/L and an additional 50% were well hydrated (280 to 295 mmol/L). Fewer than 2% of the workers demonstrated hyperosmolality (osmolality > 295 mmol/L).

Another limitation is related to the self-reported data collection of some of the risk factors including NSAID use and electrolyte intake. In an effort to minimize misclassification bias of the NSAIDs, workers were shown pictures of the over-the-counter products. NSAID use may be underreported, since Pantaleon has discouraged, but not restricted, their use. In addition, biomarkers were only collected on 3 days per worker, and we cannot exclude the possibility that workers drank more water and electrolyte solution on the study days. We were not able to follow workers after the end of the harvest to determine whether or not they eventually developed CKDu.

In conclusion, our results demonstrate that a large proportion of workers are experiencing repeat AKI, under intense heat conditions. While hydration and electrolyte intake are found to be protective, they do not prevent all injuries. This suggests that other factors may be contributing to injury that could develop into chronic damage and CKDu over time.

## Electronic supplementary material

Below is the link to the electronic supplementary material.

Supplementary material 1 (PDF 802 KB)
