# Supplementary material for: Evaluation of heat stress and cumulative incidence of acute kidney injury in sugarcane workers in Guatemala
Source: Int Arch Occup Environ Health. 2019 Apr 17;92(7):977–90. doi: 10.1007/s00420-019-01426-3 (PMC6768910; doi:10.1007/s00420-019-01426-3)
Supplement: Supplementary file 1 — Supplementary material 1 (PDF 802 KB) [file 420_2019_1426_MOESM1_ESM.pdf]

Supplemental Material

Jaime Butler-Dawson, PhD[1,2,3], Lyndsay Krisher, MPH[1,2], Hillary Yoder, MS[4], Miranda Dally, MS[1,2,3], Cecilia Sorenson, MD[2,5], Richard J. Johnson, MD[2,6], Claudia Asensio, DrPH[7], Alex Cruz, MD[7], Evan C. Johnson, PhD[4], Elizabeth J. Carlton, PhD[2,3], Liliana Tenney, MPH[1,2,3], Edwin J. Asturias, MD[8,9,10], and Lee S. Newman, MD, MA[1,2,3,11]

[1]Center for Health, Work, & Environment and the Department of Environmental and Occupational Health, Colorado School of Public Health, University of Colorado, Anschutz Medical Campus, Aurora, CO
[2]Colorado Consortium on Climate Change and Human Health, University of Colorado, Anschutz Medical Campus, Aurora, CO
[3]Department of Environmental and Occupational Health, Colorado School of Public Health, University of Colorado, Anschutz Medical Campus, Aurora, CO
[4]Department Kinesiology & Health, University of Wyoming, Laramie, WY
[5]Department of Emergency Medicine, University of Colorado School of Medicine, Anschutz Medical Campus, Aurora, CO
[6]Division of Renal Diseases and Hypertension, University of Colorado School of Medicine, Anschutz Medical Campus, Aurora, CO
[7]Pantaleon, Guatemala City, Guatemala
[8]Division of Pediatric Infectious Diseases, University of Colorado School of Medicine, Anschutz Medical Campus, Aurora, CO
[9]Center for Global Health, Colorado School of Public Health, Anschutz Medical Campus, Aurora, CO
[10]Department of Epidemiology, Colorado School of Public Health, University of Colorado, Anschutz Medical Campus, Aurora, CO
[11]Division of Pulmonary Sciences and Critical Care Medicine, Department of Medicine, University of Colorado School of Medicine, Anschutz Medical Campus, Aurora, CO

Address correspondence to Jaime Butler-Dawson, Center for Health, Work, & Environment, Colorado School of Public Health. 13001 E. 17th Pl., Ste. W3111, Aurora, CO 80045. Telephone: 1-303-724-8130. E-mail: Jaime.butler-dawson@ucdenver.edu.

I. Work setting and practices

This prospective cohort study was conducted at a sugarcane plantation privately owned by an agribusiness, Pantaleon, in southwest Guatemala in the Department of Escuintla. Work setting, worker population, and work practice details are described in a previous article (Butler-Dawson et al., 2018). Prior to the start of the annual sugarcane harvest for 2016-2017, Pantaleon recruited and screened 4,568 field workers to harvest cane from November to April. Workers were recruited from local communities in the coastal low land area surrounding the sugarcane fields as well as from the highland regions at higher altitudes. All field workers participated in a pre-employment screening prior to the start of work. The screening included a medical exam to determine if they are fit for work and to recommend a job placement. One of the hiring criteria was that workers have an estimated glomerular filtration rate, eGFR, ≥ 60 ml/min/1.73m^2 at the time of screening. Creatinine is measured from a

venipuncture blood draw and used to calculate eGFR using the Chronic Kidney Disease Epidemiology Collaboration (CKD-EPI) equation for all participants (Levey & Stevens, 2010).

The company employs two types of field workers: cane cutters and production workers. In 2016-2017, 3,621 cane cutters were hired for the harvest season. Approximately half of the cane cutters come from the local communities surrounding the plantation and half come from the highlands region. Local workers return home after the daily work shift and highland workers stay at on-site dormitories for the entire harvest and then return home to the highland region after the season is done. Cane cutters cut and pile burned sugarcane in six-day blocks and then have one rest day. They typically start their work shift around 7 am and spend ten hours in the field that includes cutting for eight hours, three 20-minute breaks, and one 60-minute lunch break. Workers receive a base wage independent of the amount of cane that is cut. Pantaleon also hired 847 field production workers for the season who perform tasks such as cutting and planting. Production workers are hired only from the local area, commute to work, spending seven hours per day in the fields, in 5-day blocks before receiving two rest days. They are compensated based on their time spent working. All workers are assigned to work groups consisting of 40 to 70 workers, each led by a team supervisor.

II. Hydration and wellness promotion program

Through its wellness program, Pantaleon encourages cane cutters to drink 16 liters of water and 2.5 liters of electrolyte solution (composition per liter: 4.6 g NaCl, 34 g carbohydrates (26 g sucrose) and 2 g KCl) per work shift and to take three 20-minute breaks and one 60-minute lunch break during the work shift. They also encourage the workers to rest in the shade that is provided. The start and finish of each break period is announced and participation monitored by field health aides and work group supervisors assigned to every work unit. The field health aides educate the workers on health topics including hydration, rest, and safety. Water and electrolyte solution are provided daily for free in the fields. Cane cutters are issued a 5-liter container that can be filled with water in tanks that are stationed in the fields. Health aides and field physicians address health issues that arise during the day. All workers have access to medical clinics staffed by company physicians and healthcare workers.

III. Pocket urine color chart for self-evaluation

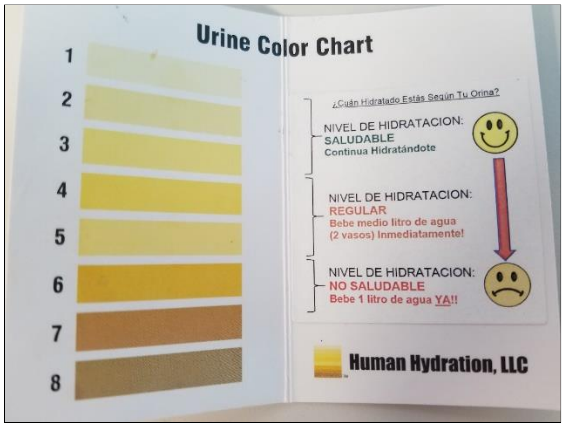

Fig. S1 *Urine color chart (Human Hydration, LLC, Hampton, VA) adapted for low literacy, Spanish speaking populations.*

IV. Clothing correction factor for post-shift body weight

To adjust for the additional weight of the clothes at the end of the day due to sweat and dirt, we weighed the clothes and shoes of 20 workers (14 cane cutters and 6 production workers) pre- and post-shift using a scale (Ranger 3000 digital scale, Ohaus, Parsippany, NJ). A correction factor was calculated by averaging the difference of the pre- and post-shift weights, separately for cane cutters and production workers (cane cutter correction factor: 244.57 grams, range -52 to 906 grams; production correction factor: 36.17, range -104 to 178 grams).

V. Characteristics of non-participants compared to participants

Table S1: Comparison of non-participants to participants by the reason for attrition, N=517.

Characteristics	Reason for Attrition		
	Left workforce early vs. did not leave early	Absent on day(s) of study vs. not absent	Refused to participate vs. workers who did not refuse
Total Number	32 (6%)	54 (10%)	10 (2%)
Age, years	**26 vs. 28, p=0.04**	32 vs. 28, p=0.66	24 vs. 28, p=0.13
Local home residence	**41% vs. 65%, p<0.01**	57% vs. 64%, p=0.31	**20% vs. 65%, p<0.01**
Cane cutter job type	88% vs 80%, p=0.33	**94% vs. 79%, p<0.01**	90% vs. 81%, p=0.46
Baseline hypertension	4% vs. 3%, p=0.76	0% vs. 4%, p=0.32	14% vs. 3%, p=0.10
Baseline eGFR	120 vs. 116, p=0.15	116 vs. 116, p=0.85	122 vs. 116, p=0.25
February eGFR	134 vs. 129, p=0.05	127 vs. 130, p=0.40	134 vs. 130, p=0.41
March eGFR	138 vs. 132, p=0.12	133 vs. 132, p=0.40	140 vs. 132, p=0.38
April eGFR	N/A	128 vs. 132, p =0.37	133 vs. 132, p=0.95

eGFR: estimated glomerular filtration rate. Bolded values are significant at p <0.05.

VI. Post-shift survey, developed in Spanish

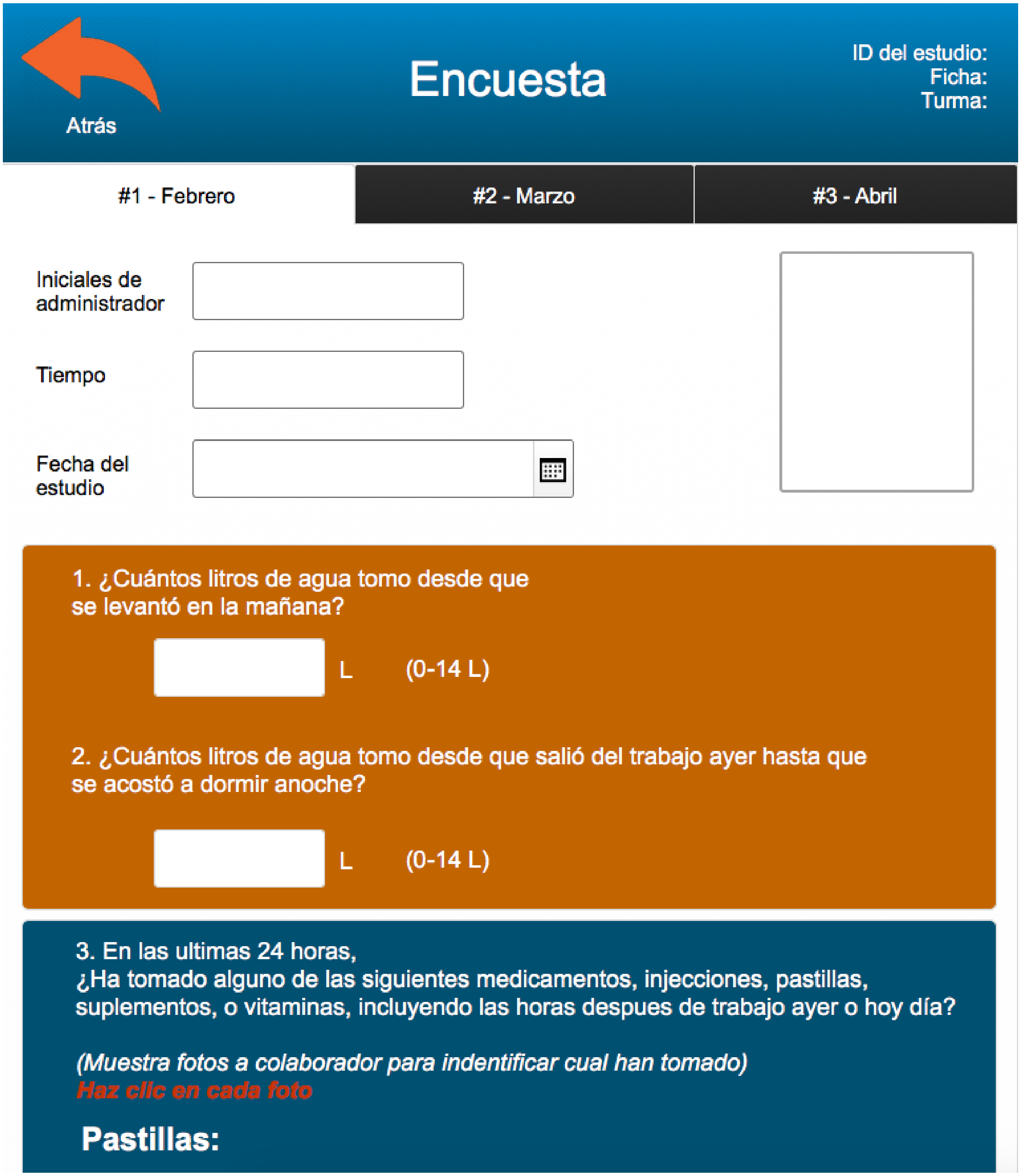

Atrás

Encuesta

ID del estudio:
Ficha:
Turma:

#1 - Febrero | #2 - Marzo | #3 - Abril

Iniciales de administrador

Tiempo

Fecha del estudio

1. ¿Cuántos litros de agua tomo desde que se levantó en la mañana?

L (0-14 L)

2. ¿Cuántos litros de agua tomo desde que salió del trabajo ayer hasta que se acostó a dormir anoche?

L (0-14 L)

3. En las ultimas 24 horas,
¿Ha tomado alguno de las siguientes medicamentos, injecciones, pastillas, suplementos, o vitaminas, incluyendo las horas despues de trabajo ayer o hoy día?

(Muestra fotos a colaborador para indentificar cual han tomado)
Haz clic en cada foto

Pastillas:

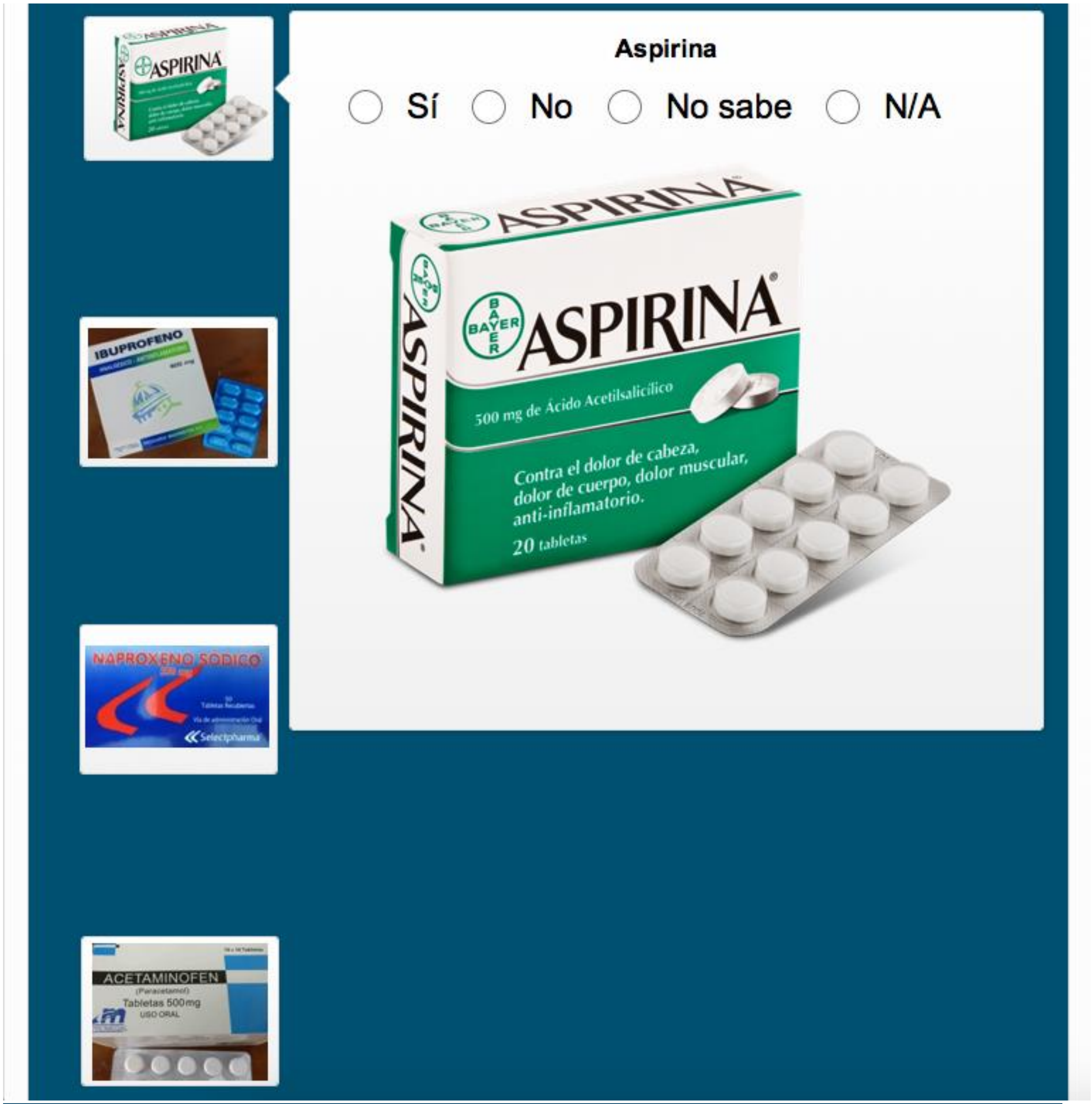
Aspirina
Sí No No sabe N/A
ASPIRINA
BAYER
500 mg de Ácido Acetilsalicílico
Contra el dolor de cabeza, dolor de cuerpo, dolor muscular, anti-inflamatorio.
20 tabletas
IBUPROFENO
NAPROXENO SODICO
Selectpharma
ACETAMINOFEN
(Paracetamol)
Tabletas 500mg
USO ORAL

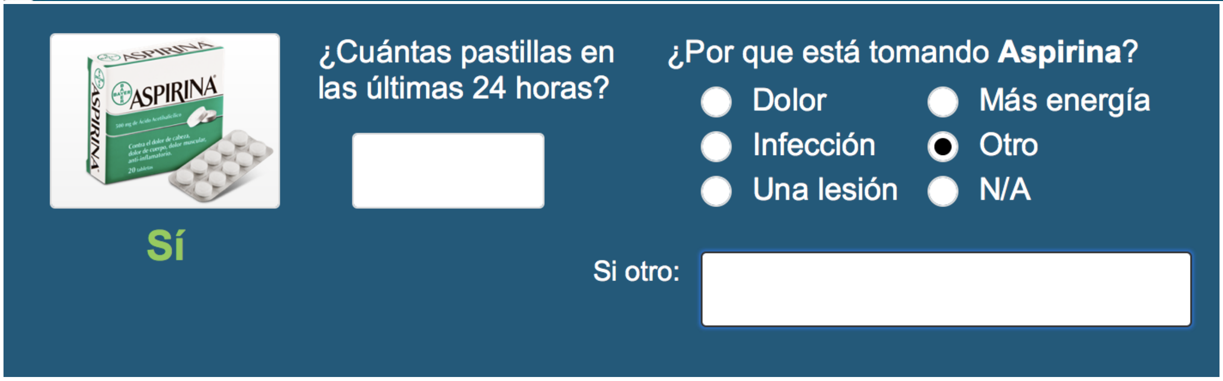
ASPIRINA
Sí
¿Cuántas pastillas en las últimas 24 horas?
¿Por que está tomando Aspirina?
Dolor
Más energía
Infección
Otro
Una lesión
N/A
Si otro:

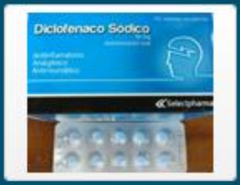
Diclofenaco Sodico
Selectpharma

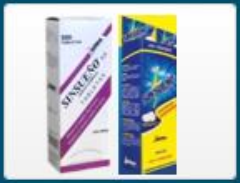
SINSUEÑO

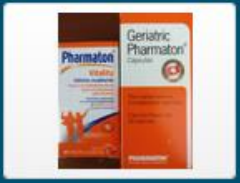
Pharmaton
Geriatric Pharmaton

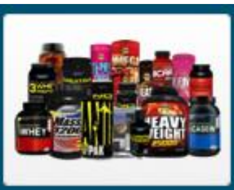

Otros

Inyecciones:

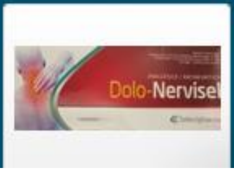

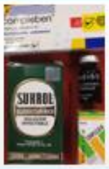

Suplementos

○ Sí ○ No ○ No sabe ○ N/A

Otros

4. ¿Cuántos cigarrillos fumo desde que se levantó esta mañana?

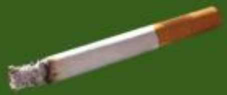

5. ¿Cuántas bolsas de suero tomo desde que se levantó esta mañana?

bolsas

7. ¿Cuántas bebidas azucaradas tomo hoy desde que se levantó esta mañana?

Por ejemplo:
jugos, Coca-Cola, Fanta, polvos para bebidas

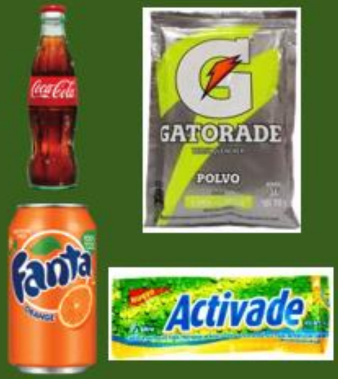

8. ¿Cuántas bebidas alcoholes tomó ayer hasta que se levantó esta mañana?

Por ejemplo:
cerveza, quetzalteca

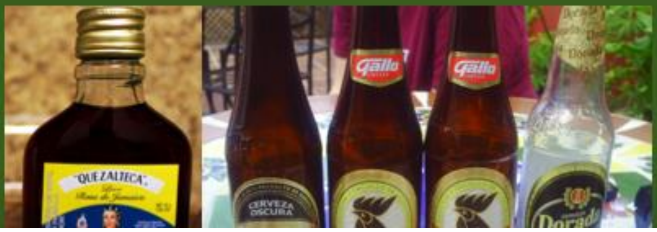

9. ¿Cuántos descansos, incluyendo el almuerzo tomo en el trabajo hoy?
(los descansos son de 15 minutos o más)

10. ¿Dónde tomo sus descansos hoy?

- Areas con sombra
- En el lugar donde me encontraba trabajando en el campo
- Ambos
- N/A

11. Hoy en el trabajo, ¿Sentiste alguno de los siguientes malestares? (selecciona todos los que apliquen)

a. Dolor de cabeza	Sí	No	No sabe	N/A
b. Calambres en las piernas o brazos	Sí	No	No sabe	N/A
c. Fiebre	Sí	No	No sabe	N/A
d. Diarrea	Sí	No	No sabe	N/A
e. Dolor al orinar	Sí	No	No sabe	N/A
f. Palpitación rapida del corazón	Sí	No	No sabe	N/A

	Sí	No	No sabe	N/A
g. Mareos	○ Sí	○ No	○ No sabe	○ N/A
h. Vomitos	○ Sí	○ No	○ No sabe	○ N/A
i. Desmayos	○ Sí	○ No	○ No sabe	○ N/A
j. Boca seca	○ Sí	○ No	○ No sabe	○ N/A
k. Dolor de oídos	○ Sí	○ No	○ No sabe	○ N/A
l. Dolor de espalda (parte de arriba)	○ Sí	○ No	○ No sabe	○ N/A
m. Dolor de espalda (parte de abajo)	○ Sí	○ No	○ No sabe	○ N/A
n. Dificultad al respirar	○ Sí	○ No	○ No sabe	○ N/A
o. Hinchazón de manos y pies	○ Sí	○ No	○ No sabe	○ N/A
p. Otro?	○ Sí	○ No	○ No sabe	○ N/A

VII. References

Butler-Dawson, J., Krisher, L., Asensio, C., Cruz, A., Tenney, L., Weitzenkamp, D., et al. (2018). Risk Factors for Declines in Kidney Function in Sugarcane Workers in Guatemala. *Journal of Occupational and Environmental Medicine*, *60*(6), 548-558.

Levey, A. S., & Stevens, L. A. (2010). Estimating GFR Using the CKD Epidemiology Collaboration (CKD-EPI) Creatinine Equation: More Accurate GFR Estimates, Lower CKD Prevalence Estimates, and Better Risk Predictions. *American Journal of Kidney Diseases*, *55*(4), 622-627.
